# Separating Risk Prediction: Myocardial Infarction vs. Ischemic Stroke in 6.2M Screenings

**DOI:** 10.3390/healthcare12202080

**Published:** 2024-10-18

**Authors:** Wonyoung Jung, Sang Hyun Park, Kyungdo Han, Su-Min Jeong, In Young Cho, Kihyung Kim, Yerim Kim, Sung Eun Kim, Dong Wook Shin

**Affiliations:** 1 Division of Cardiology, Department of Medicine, Perelman School of Medicine, University of Pennsylvania, Philadelphia, PA 19104, USA; wyjung.md@gmail.com; 2 Department of Biostatistics, College of Medicine, Catholic University of Korea, Seoul 06591, Republic of Korea; ujk8774@catholic.ac.kr; 3 Department of Statistics and Actuarial Science, Soongsil University, Seoul 06978, Republic of Korea; 4 Department of Medicine, Seoul National University College of Medicine, Seoul 03080, Republic of Korea; smjeong.fm@snu.ac.kr; 5 Department of Family Medicine and Supportive Care Center, Samsung Medical Center, Sungkyunkwan University School of Medicine, Seoul 06351, Republic of Korea; 6 Department of Digital Health, Samsung Advanced Institute for Health Science & Technology (SAIHST), Sungkyunkwan University, Seoul 16419, Republic of Korea; gckhkim@gccorp.com; 7 Department of Neurology, Kangdong Sacred Heart Hospital, Hallym University, College of Medicine, Seoul 05355, Republic of Korea; brainyrk@gmail.com; 8 Division of Cardiology, Department of Internal Medicine, Kangdong Sacred Heart Hospital, Seoul 05355, Republic of Korea; drhyangii@kdh.or.kr; 9 Department of Clinical Research Design & Evaluation, Samsung Advanced Institute for Health Science & Technology (SAIHST), Sungkyunkwan University, Seoul 16419, Republic of Korea

**Keywords:** cardiovascular disease, myocardial infarction, stroke, risk prediction, model

## Abstract

Background: Traditional cardiovascular disease risk prediction models generate a combined risk assessment for myocardial infarction (MI) and ischemic stroke (IS), which may inadequately reflect the distinct etiologies and disparate risk factors of MI and IS. We aim to develop prediction models that separately estimate the risks of MI and IS. Methods: Our analysis included 6,242,404 individuals over 40 years old who participated in a cardiovascular health screening examination in 2009. Potential predictors were selected based on a literature review and the available data. Cox proportional hazards models were used to construct 5-year risk prediction models for MI, and IS. Model performance was assessed through discrimination and calibration. Results: During a follow-up of 39,322,434.39 person-years, 89,140 individuals were diagnosed with MI and 116,259 with IS. Both models included age, sex, body mass index, smoking, alcohol consumption, physical activity, diabetes, hypertension, dyslipidemia, chronic kidney disease, and family history. Statin use was factored into the classification of dyslipidemia. The c-indices for the prediction models were 0.709 (0.707–0.712) for MI, and 0.770 (0.768–0.772) for IS. Age and hypertension exhibited a more pronounced effect on IS risk prediction than MI, whereas smoking, body mass index, dyslipidemia, and chronic kidney disease showed the opposite effect. The models calibrated well for low-risk individuals. Conclusions: Our findings underscore the necessity of tailored risk assessments for MI and IS to facilitate the early detection and accurate identification of heterogeneous at-risk populations for atherosclerotic cardiovascular disease.

## 1. Introduction

Cardiovascular disease (CVD) remains a significant global health challenge and is responsible for a substantial burden of morbidity and mortality worldwide. The number of people living with CVD worldwide nearly doubled from 1990 to 2019, contributing to a rise in disability and death from CVD [1]. Myocardial infarction (MI) and ischemic stroke (IS) are the two primary causes of CVD-related mortality [2].

MI and IS, despite their shared pathophysiology of atherosclerosis, have distinct etiologies and risk factors. However, in current clinical practice, these two conditions are often combined as atherosclerotic cardiovascular disease in risk prediction models such as the revised PCE, the Systemic Coronary Evaluation Model (SCORE) 2, the QRISK algorithm, and the PREDICT study. Modifiable risk factors, such as smoking, hypertension, diabetes, and elevated cholesterol levels, have been well established and incorporated into these models to prevent and minimize the development of a composite of MI and IS (Table 1). That approach, while valuable, might not fully capture the unique risk profiles associated with MI and IS, potentially limiting the application of prevention and management strategies. Furthermore, traditional CVD risk prediction models have primarily been developed using data from Western populations, which limits their applicability due to diversity in the incidence of CVD [3] and the distribution of cardiovascular risk factors across racial and ethnic groups [4].

Therefore, several East Asian–specific CVD risk prediction models have been developed (Table S1), specifically Korean, Japanese, and Chinese models. Among them, two Japanese models [5,6] provide separate risk scores for MI and stroke. Most of the Japanese and Chinese models are limited by small sample sizes [6,7,8,9,10,11,12], whereas all the Korean studies [13,14,15,16,17] have used large samples. However, some parameters still need to be validated [13], and the inclusion of both young adults (20–39 years) and an elderly population could require cautious interpretation due to the strong effects of age on CVD [16,17]. Moreover, except for Yoshida et al. [18], all the East Asian models still fail to include lipid-lowering medication usage. Historically, CVD risk prediction models have used total cholesterol levels to estimate CVD risk without considering the use of dyslipidemia medication [4,19,20,21]. Recently, a United Kingdom study demonstrated that including it led to moderate improvements in both calibration and discrimination [22]. Considering the pervasive use of statins and the resulting decrease in the statin-naive population, integrating their use is expected to improve the prediction accuracy of risk models.

For this study, we used a comprehensive cardiovascular health screening dataset covering more than 6 million individuals in Korea to develop distinct risk prediction models for MI and IS. Model construction was based on potential predictors identified through a review of the literature and contingent on data availability. Notably, medication for dyslipidemia is included as a predictor in these models.

## 2. Methods

### 2.1. Database Source

For model construction, we used data from the National Health Insurance Service (NHIS), a representative and population-based database in Korea. The NHIS is a universal health coverage system that provides mandatory, universal, comprehensive medical care to 97% of the Korean population, with the remaining 3% covered through the Medical Aid Program (MAP) for people with the lowest incomes. Because the NHIS also manages all administrative processes for MAP recipients, the NHIS database includes the whole Korean population.

In Korea, the NHIS initiates national general health examination programs by sending invitation letters to all national health insurance members who are older than 40 years and all employees regardless of age, at least every two years. Eligible members are then entitled to undergo a screening examination at one of several designated medical institutions, private clinics, hospitals, and public health centers that voluntarily participate in the national screening program. The purpose of this program is to identify and manage health conditions related to CVD, such as hypertension, diabetes, and dyslipidemia, at an early stage to decrease the overall impact of CVD. The program consists of a standardized questionnaire about each participant’s medical history, family history, and lifestyle behaviors such as smoking, drinking, and physical activity. That information is then combined with demographic (e.g., age, sex, and income) and anthropometric data, as well as data on prescribed medications and International Classification of Diseases, 10th revision (ICD-10) diagnostic codes from the NHIS. Once the screening is complete, participating medical institutions are obligated to report the results and provide documented feedback to the attendees before they receive reimbursement from the NHIS. If the screening finds abnormal values, individuals are advised to seek further diagnostic confirmation and relevant medical services from nearby healthcare facilities. Those medical services are primarily provided by private healthcare providers on a fee-for-service basis. Any expenses incurred during this process are subsequently reimbursed by the NHIS.

This study was approved by the Institutional Review Board of Samsung Medical Center (SMC 2022-03-106). Anonymized and de-identified information was used for the analyses; therefore, informed consent was not required.

### 2.2. Study Population

We initially identified study subjects older than 40 years (n = 7,037,730) who received a general health examination in 2009. Subjects with missing or erroneous values (n = 279,972) or any CVD history before the general health examination (n = 477,181) were sequentially excluded. After considering a 1-year lag period (n = 38,173), 6,242,404 subjects were included in the analysis (Figure S1). The development and validation datasets for the risk prediction model were generated by using simple random sampling to split the final study population, with 70% of the final study subjects selected for the development dataset and the remaining 30% used for the validation dataset. In this study, the subjects were not involved in its design, conduct, reporting, or dissemination plans.

### 2.3. Predictors

Potential predictors were selected based on a literature review and data availability. Accordingly, age, sex, BMI, smoking behavior, alcohol consumption, physical activity, diabetes, hypertension, dyslipidemia, chronic kidney disease, and family history of stroke or MI were selected. Details of classification for each predictor are shown in Table S2 and Method S1.

### 2.4. MI and IS Adjudication

Newly diagnosed MI and IS were identified using the ICD-10 codes for MI (I21 or I22 during hospitalization) and IS (I63 or I64 during hospitalization with claims for brain magnetic resonance imaging or brain computerized tomography) [23]. In this study, CVD was defined as a composite of MI and IS. The cohort was followed from the date of the health screening examination in 2009 to the date of incident CVD, censoring date (e.g., outmigration to other countries), death, or the end of the study period (31 December 2019), whichever came first.

### 2.5. Statistical Analysis

#### 2.5.1. Development of the Risk Prediction Models

Risk prediction models were developed for three outcomes: (1) MI, (2) IS, and (3) CVD. Multivariable Cox proportional hazards models were used to determine the hazard ratios and 95% confidence intervals (CIs) for the relationship between the selected predictors and the separate risks of MI and IS, as well as the overall risk of CVD. The proportional hazards assumption was assessed using Schoenfeld residuals and the logarithm of the cumulative hazard function based on Kaplan–Meier curves. The best-fit risk prediction models were developed using variables selected based on a literature review and the availability of data. The final predictors in the model were used to create a weighted-risk score by assigning scores ranging from 0 to 100 based on the beta coefficients for each predictor in the final Cox proportional hazards model. The scores were then added together to obtain a total score. This predictive model for the risk of MI and IS individually, as well as for CVD risk, was translated into a risk score nomogram that provides a numerical estimate of an individual’s risk of developing MI, IS, or CVD.

#### 2.5.2. Validation of the Risk Prediction Models

Model performance was evaluated in terms of discrimination and calibration. Discrimination was assessed using the area under the receiver operating characteristic curve (AUROC) and the concordant statistic (C-statistic), which provide information about the ability of the model to distinguish between individuals who developed the outcome (MI, IS, or CVD) and those who did not. The internal validation of discrimination was conducted using the bootstrap optimism-corrected AUROC with 100 bootstrap replications [24]. Calibration was assessed by categorizing the study subjects into deciles based on the absolute MI, IS, or CVD risk predicted by the model and comparing the predicted and observed incidence in each decile using the expected-to-observed ratio and the Hosmer-Lemeshow chi-square test. All statistical analyses were performed in SAS version 9.4 (SAS Institute Inc., Cary, NC, USA). The *p* values provided are two-sided, and the level of significance is 0.05.

## 3. Results

During a mean follow-up of 9.0 years (39,322,434.39 person-years), we identified 89,140 cases of MI, and 116,259 cases of IS. The baseline characteristics of the study population are presented in Table S3.

### 3.1. Development and Validation of MI and IS Risk Prediction Models

In the MI (Table 2) and IS (Table 3) risk prediction models, old age, being male, being underweight or obese, smoking, long-standing diabetes, uncontrolled hypertension with anti-hypertensives, untreated dyslipidemia (total cholesterol levels ≥ 240 mg/dL), a family history of CVD, and low eGFR (<30) all contributed to an increased predicted risk. However, the associations between the predictors and MI and IS differed. Smoking behavior, obesity, and dyslipidemia were more strongly associated with MI, whereas age and hypertension were more strongly associated with IS. Both models displayed a J-shaped pattern for alcohol consumption, but the risk of MI for non-drinkers was higher than the drinkers, whereas the risk of IS for heavy drinkers (≥30 g/day) was higher than for the non-drinkers.

**Figure 1:**
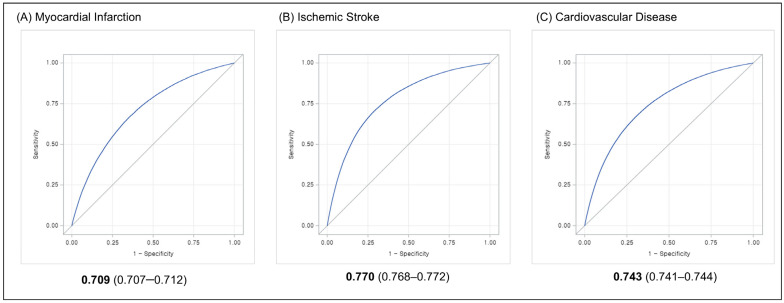
AUROC curves for the (**A**) myocardial infarction, (**B**) ischemic stroke, and (**C**) cardiovascular disease risk models.

**Figure 2:**
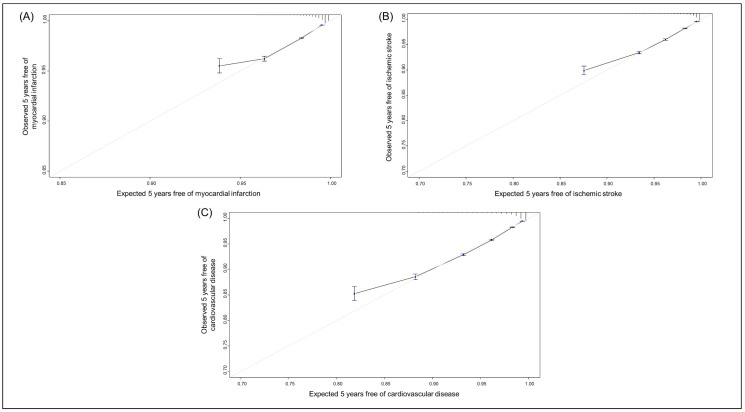
Calibration plots for the (**A**) myocardial infarction, (**B**) ischemic stroke, (**C**) cardiovascular disease risk prediction models (gray line: ideal).

The risk score nomograms for MI and IS are illustrated in Figures S2 and S3, respectively. A 5-year risk of 7.5% for MI and IS matches the risk scores of 171 and 137, respectively (Figures S4 and S5). The highest deciles of the MI and IS scores match the group with the highest incidence rates (7.3 and 12.4 per 1000 PYs, respectively) (Figure S6A,B). The AUROCs of the MI and IS risk models were 0.709 (0.707–0.712), and 0.770 (0.768–0.772), respectively (Figure 1A,B). The calibration plots demonstrate that the models performed well in predicting risk among individuals with low estimated risk but overestimated the risk of MI or IS in those with high estimated risk (Figure 2A,B).

### 3.2. Development and Validation of CVD Risk Prediction Models

All the predictors used in the MI and IS risk prediction models were retained in CVD risk prediction model (Table S3). Alcohol consumption showed a J-shaped association with CVD risk, and physical activity and the use of dyslipidemia medication showed protective effects on CVD risk.

The nomogram of the CVD risk score is shown in Figure S7. The sum of the risk score ranges from 0 (5-year CVD risk of 0.3%) to 229 (5-year CVD risk of 69.2%) and is calculated from 11 predictors. For example, a risk score of 126 matches a 5-year CVD risk of 7.5% (Figure S8). When the total CVD risk scores are categorized into deciles, individuals in the highest decile correspond with the group with the highest incidence rate, 18.7 per 1000 PYs (Figure S9). The AUROC for CVD was 0.743 (95% CI 0.741–0.744) (Figure 1C). Similarly to the MI and IS risk prediction model, the model performs well in predicting risk among individuals with low-estimated risk but overestimates the risk of CVD in those with high estimated risk (Figure 2C).

## 4. Discussion

In this development and validation study involving 6,242,404 individuals, we derived risk prediction models for MI, IS, and CVD from a comprehensive cardiovascular health screening dataset. The AUROCs of these models were 0.709 (95% CI 0.707–0.712) for MI, 0.770 (95% CI 0.768–0.772) for IS, and 0.743 (95% CI 0.741–0.744) for CVD. All three models calibrated well, especially for those with low estimated risk. Considering the different associations of predictors and CVD subtypes in the present study, and the varied distribution of cardiovascular risk factors—such as blood pressure, BMI, and fasting glucose—across various global regions [25,26,27], coupled with the identified disparities in age-standardized IS incidence rates [26], our research emphasizes the importance of strategies that assess CVD risk, tailored to both ethnicity and subtype.

Our CVD model demonstrated comparable discriminatory performance to traditional CVD risk prediction models such as the Framingham, SCORE, and QRISK models (AUROC ranged from a minimum of 0.66 to a maximum of 0.79) [28]. Moreover, our IS model showed a predictive performance comparable with those developed in East Asian studies [6,8,11,18] (Table S1). For instance, the Hisayama study [6] provided a discriminatory accuracy of 0.733 (95% CI 0.720–0.746), the Suita study [8] yielded 0.76, the China Health and Nutrition survey study [11] produced 0.74 (95% CI 0.72–0.76), and Yoshida et al. [18] reported a C-index from 0.734 to 0.748 for Cox and machine learning models (Ensemble, Elnet-Cox, and XGBoost) using data from the IQVIA Japan Claims Database. In contrast with our findings, the Hisayama study [6] and the Japanese IQVIA Claims Database study [18] reported superior discriminatory values for MI over IS. Notably, recent models from Yoshida et al. [18] and Chun et al. [29] used a large number of study participants and risk features. However, the CVD model from Yoshida requires cautious interpretation because their cohort had a lower incidence of CVD than was found in previous Japanese models, and Chun et al. developed a stroke-specific risk prediction model.

We observed that the associations between the predictors and MI and IS differed in the risk prediction models: age and hypertension exhibited a more pronounced effect on IS risk prediction than MI prediction, whereas smoking, BMI, dyslipidemia, and chronic kidney disease showed the opposite effect. This parallels the Japanese risk scores, which also identify age and hypertension as primary contributors to IS risk and smoking and non-HDL cholesterol as key predictors of MI [5]. Atherosclerosis, a complex and multifactorial process, underlies both MI and IS. However, MI and IS have different etiologies: MI is primarily due to plaque rupture and in situ thrombosis (90–95%), whereas IS has multifactorial causes. The incidence of atrial fibrillation, a well-established risk factor for IS, increases with age, which could explain our finding of a more prominent association between old age and IS risk than MI risk.

Alcohol consumption also had different effects on the risks of MI and IS. Mild-to-moderate alcohol consumption was associated with a reduced risk of IS, whereas heavy drinking was linked to an increased risk. This J-shaped pattern is generally consistent with prior studies. On the other hand, MI risk was lower for alcohol drinkers than non-drinkers even at higher doses.

Intriguingly, the use of dyslipidemia medication was associated with lower risks of CVD, MI, and IS than untreated hypercholesterolemia. This aligns with the methodological assertion of Xu et al. [22], who emphasized the need to consider future statin use when predicting CVD risk. Current CVD prevention guidelines emphasize the initiation of statins for preventing MI and IS, but they overlook the role of current dyslipidemia medication in predicting future CVD risk. Given the compelling evidence of dyslipidemia medications’ risk-reducing effects on CVD, future research should include them in risk assessments for CVD.

Family history, a well-established cardiovascular risk factor, was unexpectedly found to contribute minimally to the risk of MI and IS in our study. That might be because we gathered family history data through self-reported questionnaires, which might yield unreliable information. Furthermore, the questionnaire did not collect information about onset age for the familial CVD history. The number of family members whose CVD history was requested might also partially account for our findings.

The robustness of our models is assured by our large sample of 6,242,404 individuals with complete CVD risk assessment data from a national screening examination. The national health screening program in Korea facilitates the early detection and timely management of modifiable risk factors such as blood pressure, cholesterol, and blood glucose levels, with the aim of preventing progression to higher risk categories and potentially reducing the burden of CVD at the population level. Furthermore, the NHIS provides follow-up educational counseling and treatment referrals for individuals with these health conditions. Given that CVD typically develops over decades, health risk assessments can serve as a preventive measure against the onset of CVD. Stemming from health screening settings, our models could promise timely management of cardiovascular risk factors, empowering clinicians to offer informed counseling. Continued progress in this field could potentially lessen the global burden of CVD.

Despite the strengths and notable clinical implications of this study, it is important to recognize its limitations. First, the database used for this study was collected from the Korean population, which could limit the generalizability of the findings to other ethnic groups, so caution should be taken when extrapolating the results. Second, our models overestimated CVD, MI, and IS risk in high-risk individuals. Potential factors in that finding include model development using a predominantly low-risk population, which could limit generalizability to high-risk individuals. For example, the prevalence of cardiometabolic risk factors in Korea is lower than in the US [30]. Third, although the internal validation of our models was thorough, external validation is still needed. Research has shown that only 36% of CVD risk prediction models have been externally validated [28]. Given that our models are based on a nationwide population database encompassing more than 6 million individuals, the need for further validation of our findings, particularly using different outcome definitions, is highlighted. Finally, some risk factors, such as genetic data or inflammatory markers, were not incorporated into the model.

In conclusion, our findings highlight the critical importance of tailored risk assessments for MI and IS, aiming for early detection and precise identification of heterogeneous at-risk populations for atherosclerotic cardiovascular disease (e.g., those at high risk for MI but low risk for IS, or vice versa). By identifying individuals with elevated estimated risks of MI and IS, healthcare providers can implement targeted preventive interventions such as statin therapy.

## Figures and Tables

**Table 1:** Comparison of predictors in cardiovascular disease risk models.

	Framingham CHD	NCEP-ATP III	Framingham Global	Reynolds	QRISK2	Pooled Cohort Equation	Revised Framingham Stroke	QRISK3
	1998	2001	2008	2008	2008	2013	2017	2017
Age	O	O	O	O	O	O	O	O
Sex	O	O	O		O	O	O	O
Race					O	O		O
Townsend deprivation score *					O			O
Total cholesterol	O	O	O			O	O	
LDL cholesterol	O							
HDL cholesterol	O	O	O	O		O		
Total/HDL cholesterol ratio					O			O
Systolic BP	O	O	O	O	O	O	O	O
Antihypertensive treatment		O	O		O	O	O	O
Diabetes	O		O		O	O	O	O
HbA1c				O (female)				
Atrial fibrillation					O		O	O
Smoking	O	O	O	O	O	O	O	O
Previous CVD							O	
Family history				O	O (before age 60)			O (before age 60)
CKD					O (stage 4, 5)			O (stage 3, 4, 5)
Rheumatoid arthritis					O			O
CRP				O				O
Systolic BP variability								O
Migraine								O
Corticosteroid use								O
SLE								O
Atypical antipsychotics								O
Severe mental illness								O
Erectile dysfunction								O

LDL, low-density lipoprotein; HDL, high-density lipoprotein; BP, blood pressure; CVD, cardiovascular disease; CKD, chronic kidney disease; CRP, C-reactive protein; SLE, systemic lupus erythematosus. * Measure incorporates four variables: unemployment, non-car ownership, non-home ownership, household overcrowding.

**Table 2:** Predictors of myocardial infarction in the risk prediction model.

	Subjects (N)	Cases (n)	IR	Crude Model HR (95% CI)	Final Model aHR (95% CI)	Point
Age, years						
40–44	958,910	7730	0.87	1 (Ref.)	1 (Ref.)	0
45–49	801,224	9280	1.25	1.44 (1.39–1.48)	1.36 (1.31–1.40)	13
50–54	862,220	13,271	1.67	1.91 (1.86–1.96)	1.72 (1.67–1.77)	24
55–59	511,152	10,546	2.26	2.58 (2.51–2.66)	2.15 (2.08–2.21)	34
60–64	495,753	13,055	2.90	3.31 (3.22–3.41)	2.64 (2.57–2.72)	43
65–69	329,690	12,259	4.19	4.82 (4.68–4.96)	3.69 (3.58–3.80)	57
70–74	267,354	13,568	5.93	6.88 (6.69–7.07)	5.19 (5.03–5.34)	72
75–79	92,800	5978	8.13	9.65 (9.33–9.98)	7.29 (7.04–7.56)	87
≥80	50,580	3453	10.16	12.68 (12.18–13.19)	9.60 (9.21–10.02)	100
Male, sex	2,164,819	51,078	2.63	1.41 (1.39–1.43)	1.39 (1.36–1.41)	14
Female, sex	2,204,864	38,062	1.88	1 (Ref.)	1 (Ref.)	0
Body mass index, kg/m^2^						
<18.5	99,200	2496	2.93	1.52 (1.46–1.58)	1.30 (1.25–1.35)	11
18.5–23	1,598,569	28,331	1.96	1 (Ref.)	1 (Ref.)	0
23–25	1,163,819	23,416	2.21	1.13 (1.11–1.15)	1.02 (1.00–1.04)	1
25–30	1,366,503	31,249	2.51	1.28 (1.26–1.30)	1.08 (1.06–1.10)	3
≥30	141,592	3648	2.83	1.45 (1.40–1.50)	1.20 (1.16–1.25)	8
Smoking behavior						
Non-smoker	2,807,429	51,349	2.00	1 (Ref.)	1 (Ref.)	0
Mild (<20 PY)	806,675	15,096	2.06	1.04 (1.02–1.06)	1.27 (1.24–1.30)	10
Moderate (≥20 PY, <40 PY)	586,338	15,590	2.97	1.49 (1.47–1.52)	1.59 (1.56–1.63)	20
Heavy (≥40 PY)	169,241	7105	4.89	2.48 (2.42–2.54)	1.71 (1.66–1.76)	24
Alcohol consumption						
Non-drinker	2,540,147	56,070	2.43	1 (Ref.)	1 (Ref.)	11
Mild (<15 g/day)	1,071,056	18,257	1.87	0.77 (0.76–0.78)	0.82 (0.81–0.84)	3
Moderate (≥15 g/day, <30 g/day)	435,462	7964	2.01	0.83 (0.81–0.85)	0.77 (0.75–0.79)	0
Heavy (≥30 g/day)	323,018	6849	2.36	0.98 (0.95–1.00)	0.80 (0.78–0.82)	1
Physical activity						
None	2,193,805	50,783	2.57	1 (Ref.)	1 (Ref.)	8
Insufficient	1,302,812	21,614	1.81	0.70 (0.69–0.72)	0.86 (0.85–0.88)	2
Sufficient	873,066	16,743	2.10	0.82 (0.80–0.83)	0.83 (0.82–0.85)	0
Diabetes						
Normal (FPG < 100 mg/dL)	2,786,335	48,232	1.89	1 (Ref.)	1 (Ref.)	0
Prediabetes (100 ≤ FPG < 126 mg/dL)	1,109,425	22,403	2.23	1.18 (1.16–1.20)	0.99 (0.98–1.01)	0
New-onset diabetes (FPG ≥ 126 mg/dL)	155,778	4348	3.15	1.68 (1.63–1.73)	1.26 (1.22–1.30)	11
Recent onset diabetes (<5 years)	166,884	6202	4.21	2.23 (2.18–2.29)	1.39 (1.35–1.43)	15
Long-standing diabetes (≥5 years)	151,261	7955	6.11	3.27 (3.19–3.35)	1.80 (1.76–1.85)	26
Hypertension						
Normal	1,312,777	16,850	1.40	1 (Ref.)	1 (Ref.)	0
Prehypertension	1,606,473	27,062	1.84	1.32 (1.30–1.35)	1.10 (1.08–1.13)	4
New-onset hypertension	412,133	9480	2.56	1.84 (1.80–1.89)	1.29 (1.26–1.32)	11
Controlled with anti-hypertensives	683,978	23,248	3.82	2.75 (2.70–2.81)	1.40 (1.37–1.43)	15
Uncontrolled with anti-hypertensives	354,322	12,500	3.98	2.86 (2.80–2.93)	1.42 (1.38–1.45)	15
Dyslipidemia						
Untreated, TC < 200 mg/dL	2,088,585	35,958	1.90	1 (Ref.)	1 (Ref.)	0
Untreated, 200 mg/dL ≤ TC < 240 mg/dL	1,335,630	26,519	2.18	1.15 (1.13–1.16)	1.14 (1.12–1.16)	6
Untreated, 240 mg/dL ≤ TC	427,532	10,782	2.78	1.47 (1.44–1.50)	1.43 (1.40–1.47)	16
Treated with lipid-lowering agent	517,936	15,881	3.39	1.78 (1.75–1.81)	1.16 (1.13–1.18)	6
CKD (Estimated GFR)						
<30	5042	362	9.21	4.50 (4.06–4.99)	2.62 (2.36–2.91)	47
30–60	248,392	9419	4.34	2.07 (2.02–2.11)	1.18 (1.16–1.21)	7
≥60	4,116,249	79,359	2.12	1 (Ref.)	1 (Ref.)	0
Family history of stroke or MI						
No	3,969,283	81,655	2.27	1 (Ref.)	1 (Ref.)	0
Yes	400,400	7485	2.04	0.90 (0.88–0.92)	1.02 (1.00–1.05)	1

HR, hazard ratio; aHR, adjusted hazard ratio; PY, pack*year; FPG, fasting plasma glucose; TC, total cholesterol; CKD, chronic kidney disease; GFR, glomerular filtration rate; MI, myocardial infarction.

**Table 3:** Predictors of ischemic stroke in the risk prediction model.

	Subjects (N)	Cases (n)	IR	Crude Model HR (95% CI)	Final Model aHR (95% CI)	Point
Age, years						
40–44	958,910	5904	0.67	1 (Ref.)	1 (Ref.)	0
45–49	801,224	8187	1.11	1.66 (1.61–1.72)	1.57 (1.52–1.62)	15
50–54	862,220	13,138	1.65	2.48 (2.41–2.56)	2.25 (2.19–2.33)	27
55–59	511,152	12,348	2.65	3.98 (3.86–4.10)	3.36 (3.25–3.46)	40
60–64	495,753	17,921	3.99	6.01 (5.84–6.19)	4.85 (4.71- 5.00)	52
65–69	329,690	19,271	6.65	10.04 (9.75–10.34)	7.73 (7.50–7.97)	68
70–74	267,354	23,093	10.24	15.58 (15.14–16.03)	11.74 (11.39–12.10)	81
75–79	92,800	10,557	14.64	22.59 (21.88–23.32)	16.94 (16.38–17.51)	93
≥80	50,580	5840	17.52	27.85 (26.86–28.88)	20.63 (19.87–21.43)	100
Male, sex	2,164,819	64,855	3.35	1.32 (1.30–1.34)	1.30 (1.28–1.32)	9
Female, sex	2,204,864	51,404	2.55	1 (Ref.)	1 (Ref.)	0
Body mass index, kg/m^2^						
<18.5	99,200	3191	3.76	1.41 (1.36–1.46)	1.12 (1.08–1.16)	5
18.5–23	1,598,569	38,926	2.69	1 (Ref.)	1 (Ref.)	1
23–25	1,163,819	30,452	2.88	1.07 (1.05–1.08)	0.96 (0.95–0.97)	0
25–30	1,366,503	39,327	3.17	1.17 (1.16–1.19)	0.97 (0.96–0.99)	0
≥30	141,592	4363	3.40	1.26 (1.22–1.30)	1.03 (0.99–1.06)	2
Smoking behavior						
Non-smoker	2,807,429	70,785	2.77	1 (Ref.)	1 (Ref.)	0
Mild (<20 PY)	806,675	17,575	2.40	0.87 (0.86–0.89)	1.13 (1.11–1.15)	4
Moderate (≥20 PY, <40 PY)	586,338	17,966	3.43	1.24 (1.22–1.26)	1.33 (1.31–1.36)	9
Heavy (≥40 PY)	169,241	9933	6.88	2.51 (2.46–2.56)	1.48 (1.45–1.52)	13
Alcohol consumption						
Non-drinker	2,540,147	72,518	3.16	1 (Ref.)	1 (Ref.)	2
Mild (<15 g/day)	1,071,056	22,922	2.35	0.75 (0.73–0.76)	0.95 (0.94–0.97)	0
Moderate (≥15 g/day, <30 g/day)	435,462	10,811	2.74	0.87 (0.85–0.89)	1.03 (1.01–1.05)	3
Heavy (≥30 g/day)	323,018	10,008	3.46	1.10 (1.08–1.12)	1.12 (1.09–1.14)	5
Physical activity						
None	2,193,805	68,580	3.48	1 (Ref.)	1 (Ref.)	7
Insufficient	1,302,812	26,129	2.19	0.63 (0.62–0.64)	0.86 (0.85–0.87)	2
Sufficient	873,066	21,550	2.71	0.78 (0.77–0.79)	0.81 (0.80–0.82)	0
Diabetes						
Normal (FPG < 100 mg/dL)	2,786,335	60,126	2.37	1 (Ref.)	1 (Ref.)	0
Prediabetes (100 ≤ FPG < 126 mg/dL)	1,109,425	29,250	2.92	1.23 (1.22–1.25)	1.01 (0.99–1.02)	0
New-onset diabetes (FPG ≥ 126 mg/dL)	155,778	6349	4.62	1.96 (1.91–2.02)	1.42 (1.39–1.46)	12
Recent onset diabetes (<5 years)	166,884	9013	6.16	2.61 (2.56–2.67)	1.52 (1.49–1.56)	14
Long-standing diabetes (≥5 years)	151,261	11,521	8.93	3.81 (3.73–3.89)	1.87 (1.83–1.91)	21
Hypertension						
Normal	1,312,777	17,108	1.42	1 (Ref.)	1 (Ref.)	0
Prehypertension	1,606,473	32,494	2.22	1.57 (1.54–1.60)	1.26 (1.23–1.28)	7
New-onset hypertension	412,133	14,518	3.94	2.79 (2.73–2.85)	1.75 (1.71–1.79)	19
Controlled with anti-hypertensives	683,978	32,572	5.38	3.81 (3.74–3.88)	1.61 (1.58–1.65)	16
Uncontrolled with anti-hypertensives	354,322	19,567	6.28	4.45 (4.36–4.54)	1.80 (1.76–1.84)	19
Dyslipidemia						
Untreated, TC < 200mg/dL	2,088,585	49,990	2.65	1 (Ref.)	1 (Ref.)	2
Untreated, 200 mg/dL ≤ TC < 240 mg/dL	1,335,630	33,856	2.79	1.05 (1.04–1.07)	1.05 (1.03–1.06)	3
Untreated, 240 mg/dL ≤ TC	427,532	12,376	3.20	1.21 (1.19–1.23)	1.17 (1.15–1.19)	7
Treated with lipid-lowering agent	517,936	20,037	4.29	1.62 (1.59–1.64)	0.95 (0.93–0.97)	0
CKD (Estimated GFR)						
<30	5042	405	10.36	3.91 (3.55–4.31)	1.98 (1.80–2.18)	23
30–60	248,392	14,258	6.63	2.45 (2.41–2.49)	1.20 (1.17–1.22)	6
≥60	4,116,249	101,596	2.72	1 (Ref.)	1 (Ref.)	0
Family history of stroke or MI						
No	3,969,283	106,956	2.98	1 (Ref.)	1 (Ref.)	0
Yes	400,400	9303	2.54	0.85 (0.84–0.87)	1.05 (1.03–1.08)	2

HR, hazard ratio; aHR, adjusted hazard ratio; PY, pack × year; FPG, fasting plasma glucose; TC, total cholesterol; CKD, chronic kidney disease; GFR, glomerular filtration rate; MI, myocardial infarction.

## Data Availability

The database is open to all researchers whose study protocols are approved by the official review committee.

## Supplementary Materials

The following supporting information can be downloaded at: https://www.mdpi.com/article/10.3390/healthcare12202080/s1. Figure S1. Flowchart of the study population; Figure S2. Nomogram for 5-year prediction model for myocardial infarction; Figure S3. Nomogram for 5-year prediction model for ischemic stroke; Figure S4. 5-year myocardial infarction incidence probability (%); Figure S5. 5-year ischemic stroke incidence probability (%); Figure S6. Predicted 5-year (A) myocardial infarction, and (B) ischemic stroke incidence rates (IRs) (per 1000 person-years (PYs) based on decile risk scores in the development and validation cohorts; Figure S7. Nomogram for 5-year prediction model for cardiovascular disease; Figure S8. 5-year cardiovascular disease incidence probability (%); Figure S9. Predicted 5-year cardiovascular disease incidence rates (IRs) (per 1000 person-years (PYs) based on decile risk scores in the development and validation cohorts. Table S1. Summary of previous risk prediction models for cardiovascular disease in the Asian population. Table S2. Categories and Their Respective Divisions for Predictors Used in This Study. Table S3. Baseline characteristics of the study population in the development and validation cohorts. Table S4. Predictors for cardiovascular disease in the risk prediction model. Method S1. Classification of each predictors. Refs. [31,32,33,34] are cited in the Supplementary Materials.

## Abbreviations

aHR	adjusted hazard ratio
CI	confidence interval
CVD	cardiovascular disease
DM	diabetes mellitus
IS	ischemic stroke
MI	myocardial infarction
NHIS	National Health Insurance Service
