# Supplementary material for: Separating Risk Prediction: Myocardial Infarction vs. Ischemic Stroke in 6.2M Screenings
Source: Healthcare (Basel). 2024 Oct 18;12(20):2080. doi: 10.3390/healthcare12202080 (PMC11507110; doi:10.3390/healthcare12202080)
Supplement: Supplementary file 1 [file healthcare-12-02080-s001.zip › healthcare-3165368-Supplement.pdf]

TABLE OF CONTENTS

SECTION S1. SUPPLEMENTAL TABLES

eTable S1. Summary of previous risk prediction models for cardiovascular disease in the Asian population

eTable S2. Categories and Their Respective Divisions for Predictors Used in This Study

eTable S3. Baseline characteristics of the study population in the development and validation cohorts

eTable S4. Predictors for cardiovascular disease in the risk prediction model

SECTION S2. Supplemental Methods

eMethod S1. Classification of each predictors

SECTION S3. eReference

eTable S1. Summary of previous risk prediction models for cardiovascular disease in the East Asian population

Model/Study (Author et al.)*	Year	Target population	Sample size	n-year risk	Risk factors	Discriminatory accuracy (AUC)	Significance
**The KNHS study (Choi)** [20]	2021	Korea, 40-79 years	21,581,796	8	Age, SBP, DBP, WC, fasting glucose, GGT, total cholesterol, HDL-C, LDL-C, TG, eGFR, proteinuria, BMI, smoking, alcohol drinking, physical activity, medication for diabetes, hypertension, dyslipidemia, family history of heart disease, family history of stroke	Men: **0.762** (0.760-0.764)<br>Women: **0.811** (0.809-0.813)	Sex-specific 8-year risk
**The IQVIA claim study (Yoshida)** [25]	2023	Japan, 30-74 years	572,971	N/A	Age, sex, height, weight, BMI, abdominal circumference, HbA1c, fasting glucose, hematocrit, HDL-C, LDL-C, TG, SBP, DBP, AST, ALT, r-GTP, uric acid, creatinine, smoking, eating speed, late supper, eating snacks/sweets, skipping breakfast, drinking habit, drinking amount, sleep, walking, walking pace, exercise, weight change from last year, weight change from age 20, history of CKD and hemodialysis, antihypertensive medication, diabetic medication, dyslipidemia medication, willingness to change habits	Imputed model<br>1) Ensemble<br>IHD: **0.896**<br>Stroke: **0.745**<br><br>2) Elnet-Cox<br>IHD: **0.894**<br>Stroke: **0.734**<br><br>3) XGBoost<br>IHD: **0.893**<br>Stroke: **0.737**<br><br>4) Cox model<br>IHD: **0.894**<br>Stroke: **0.748**	Integrated a large number of features in the development process.<br><br>Lower incidence of IHD and stroke in the study cohort than in previous studies in Japan is noted.
**The Hisayama study (Honda)** [26]	2022	Japan, 40-84 years	2,454	10	Age, sex, SBP, diabetes, HDL-C, LDL-C, proteinuria, smoking, regular exercise	**0.789**	New prediction model for the summated risk of ASCVD events defined as CHD or atherothrombotic brain infarction using data on the reliable diagnosis of stroke subtypes

**The JALS study (Harada)** [27]	2019	Japan<br>(40-89 years)	67,969	5-, 10-	HDL-C, blood pressure (with SBP and medication), age, non-HDL-C, diabetes, sex, current smoker, eGFR, atrial fibrillation	CVD:<br>1) **0.764** (-AF)<br>2) **0.771** (+AF)<br><br>MI<br>1) **0.812** (-AF)<br>2) **0.814** (+AF)<br><br>Stroke<br>1) **0.764** (-AF)<br>2) **0.772** (+AF)	eGFR was included in MI, and stroke but excluded in CVD.<br><br>Atrial fibrillation was excluded in model 1) and included in model 2).
**The Hisayama study (Honda)** [28]	2018	Japan<br>(40-84 years)	2,462	10	Age, sex, SBP, diabetes, current smoker<br><br>Stroke: regular exercise<br>CHD: HDL-C, LDL-C	CVD: 0.743<br>(0.732-0.754)<br><br>**Stroke: 0.733**<br>**(0.720-0.746)**<br><br>CHD: 0.784<br>(0.770-0.798)	
**The China-PAR project (Yang)** [31]	2016	China,<br>35-74 years	21,320	10	Age, SBP, total cholesterol, HDL-C, current smoking, diabetes<br>- Men: WC, geographic region, urbanization, family history of ASCVD<br>- Women: WC and geographic region	Men: **0.794**<br>(0.775-0.814)<br>Women: **0.811**<br>(0.787-0.835)	Sex-specific 10-year risk
**Myocardial infarction**							
**The Korean CHD risk model (KHS) (Jee)** [21]	2014	Korea,<br>30-74 years	268,315	10	Age, BP, total cholesterol, smoking, diabetes, HDL-C, LDL-C, TG	Men: **0.764**<br>(0.752-0.774)<br>Women: **0.815**<br>(0.795-0.835)	When compared with the Korean CHD risk model, the FRS overestimated the risk of CHD in the Korean population, where CHD incidence is relatively low.
**Stroke**							
**KCPS-II Biobank (Jung)** [22]	2018	Korea,<br>43.1±9.3 years	156,701	6	Age, sex, hypertension, diabetes, dyslipidemia, smoking status<br><br>16 SNPs	**1) Multiple logistic model**<br>**T: 0.79**<br>I: 0.83<br>H: 0.68<br><br>2) Cox proportional hazard model	Traditional and genetic risk scores were evaluated in both a multiple logistic model and a Cox proportional hazard model.

						T: 0.75<br>G: 0.63<br>Both T, G: 0.78	
**The KNHIS (Lee)** [23]	2017	Korea<br>(≥20 years)	5,715,311<br>(3,182,325 men,<br>2,532,986<br>women)	10	Age, history of hypertension, history of heart disease, history of stroke, family history of heart disease, exercise, alcohol drinking, total cholesterol, smoking, hypertension, diabetes, BMI<br><br>Only for women: family history of stroke	Men: 0.83<br>(0.82-0.84)<br><br>Women: 0.82<br>(0.81-0.83)	Sex-specific 10-year risk
**KSRP model (Jee)** [24]	2008	Korea,<br>30-84 years	1,223,740	10	Age, SBP, diabetes, total cholesterol, smoking, physical activity, BMI, alcohol	Men: **0.8165**<br>(0.7993-0.8337)<br>Women: **0.8095**<br>(0.7875-0.8315)	Sex-specific 10-year risk
**The Suita study (Arafa)** [29]	2022	Japan,<br>(30-79 years)	6,641	17.1	Age, current smoking, increased BP, impaired fasting glucose, diabetes, chronic kidney disease, atrial fibrillation	0.76	
**The Japan public health center study (Yatsuya)** [30]	2013	Japan<br>(40-69 years)	15,672	10	Age, sex, current smoking, BMI, blood pressure, antihypertensive medication use, diabetes	0.74<br>(Cox)<br><br>0.73<br>(point-based)	
**The China Health and Nutrition survey (Yu)** [32]	2021	China<br>(45-80 years)	3,124	6	Total: age, sex, hypertension, total cholesterol<br><br>Ischemic: age, sex, hypertension, LDL-C<br><br>Hemorrhagic: age, sex, hypertension, BMI, HDL-C	Total<br>0.74 (0.72-0.76)<br>Ischemic<br>0.74 (0.71-0.77)<br>Hemorrhagic<br>0.81 (0.78-0.84)	
**China Kadoorie Biobank (Chun)** [33]	2021	China<br>(30-79 years)	512,726	9	Age, current smoking, history of coronary heart disease, diabetes, BP-lowering drug use, SBP, geographical region<br><br>* additional risk factors in expanded model (men 66, women 70 factors) using LASSO regularization	* FSRP model<br>Men: 0.78<br>(0.77-0.79)<br>Women: 0.77 (0.76-0.78)<br><br>* Expanded model<br>Men: 0.83	Ischemic stroke<br>Men<br>F: 0.77 (0.76-0.78)<br>E: 0.83 (0.82-0.84)<br><br>Women<br>F: 0.76 (0.76-0.77)<br>E: 0.83 (0.82-0.84)

						(0.82-0.84)<br>Women: 0.83<br>(0.82-0.84)	Hemorrhagic stroke<br>Men<br>F: 0.79 (0.78-0.81)<br>E: 0.82 (0.81-0.84)<br><br>Women<br>F: 0.78 (0.76-0.80)<br>E: 0.82 (0.80-0.84)
**The China-PAR project (Xing)** [34]	2019	China<br>(≥55 years)	21,320	10	Age, treated/untreated SBP, current smoking, total cholesterol<br>Men: HDL-C, geographic region, urbanization, parental history of stroke<br>Women: HDL-C, waist circumference, geographic region, urbanization	Men: 0.810<br>(0.787-0.833)<br>Women: 0.810<br>(0.783-0.837)	Sex-specific 10-year risk

[*]Numbers are consistent with the reference number from the main text.

IQVIA, IMS, Quintiles, and VIA; GGT, gamma-glutamyl transferase; TG, triglycerides; eGFR, estimated glomerular filtration rate; KHS, Korean Heart Study; FRS, Framingham risk score; BMI, body mass index; JALS, Japan Atherosclerosis Longitudinal Study; KCPS-II, the Korean Cancer Prevention Study-II; SNPs, single nucleotide polymorphisms; I, ischemic stroke; H, hemorrhagic stroke; T, Traditional risk score; G, genetic risk score; KSRP, Korean Stroke Risk Prediction

eTable S2. Categories and Their Respective Divisions for Predictors Used in This Study

**Age (years)**	40-44, 45-49, 50-54, 55-59, 60-64, 65-69, 70-74, 75-79, ≥80
**Sex**	Male, Female
**BMI (kg/m^2)**	<18.5, 18.5-23, 23-25, 25-30, ≥30
**Smoking Behavior**	Non-smoker, Mild (<20 PY), Moderate (≥20PY, <40PY), Heavy (≥40 PY)
**Alcohol Consumption**	Non-drinker, Mild (<15g/day), Moderate (≥15g/day, <30g/day), Heavy (≥30g/day)
**Physical Activity**	None, insufficient, Sufficient
**Diabetes Mellitus**	Normal (FPG<100), Prediabetes (100≤FPG<126), New-onset diabetes (FPG≥126, undiagnosed), Recent onset (<5 years), Long-standing (≥5 years)
**Hypertension**	Normal, Prehypertension, New-onset hypertension, Controlled with anti-hypertensives, Uncontrolled with anti-hypertensives
**Dyslipidemia**	Untreated, TC<200; Untreated, 200≤TC<240; Untreated, TC≥240; **Treated** with lipid-lowering agent
**Chronic Kidney Disease (eGFR)**	<30, 30-60, ≥60
**Family history of stroke or MI**	No, Yes

eTable S3 Baseline characteristics of the study population in the development and validation cohorts (all P<0.001)

	Development Cohort (n=4,369,683)		Validation Cohort (n=1,872,721)	
	Non-CVD (n=4,173,987)	CVD (n=195,696)	Non-CVD (n=1,788,793)	CVD (n=83,928)
Age, years	53.35±10.01	62.04±10.97	53.36±10.02	62±11.01
40-44	945,584 (22.7)	13,326 (6.8)	405,372 (22.7)	5,775 (6.9)
45-49	784,206 (18.8)	17,018 (8.7)	336,902 (18.8)	7,255 (8.6)
50-54	836,597 (20.0)	25,623 (13.1)	357,221 (20.0)	11,217 (13.4)
55-59	489,140 (11.7)	22,012 (11.3)	209,160 (11.7)	9,410 (11.2)
60-64	466,129 (11.2)	29,624 (15.1)	199,905 (11.2)	12,627 (15.1)
65-69	299,887 (7.2)	29,803 (15.2)	129,088 (7.2)	12,678 (15.1)
70-74	233,012 (5.6)	34,342 (17.6)	99,456 (5.6)	14,509 (17.3)
75-79	77,491 (1.9)	15,309 (7.8)	33,540 (1.9)	6,731 (8.0)
≥80	41,941 (1.0)	8,639 (4.4)	18,149 (1.0)	3,726 (4.4)
Male, sex	2,054,328 (49.2)	110,491 (56.5)	881,615 (49.3)	47,392 (56.5)
Body mass index, kg/m^2	23.93±3.02	24.16±3.15	23.93±3.02	24.18±3.16
<18.5	93,829 (2.3)	5,371 (2.7)	40,183 (2.3)	2,251 (2.7)
18.5-23	1,534,581 (36.8)	63,988 (32.7)	657,073 (36.7)	27,421 (32.7)
23-25	1,112,430 (26.7)	51,389 (26.3)	476,724 (26.7)	22,012 (26.2)
25-30	1,299,188 (31.1)	67,315 (34.4)	557,043 (31.1)	28,896 (34.4)
≥30	133,959 (3.2)	7,633 (3.9)	57,770 (3.2)	3,348 (4.0)
Smoking behavior				
Non-smoker	2,691,143 (64.5)	116,286 (59.4)	1,152,941 (64.5)	49,827 (59.4)
Mild (<20 PY)	775,477 (18.6)	31,198 (15.9)	331,912 (18.6)	13,377 (15.9)
Moderate (≥20 PY, <40 PY)	554,222 (13.3)	32,116 (16.4)	238,018 (13.3)	13,709 (16.3)
Heavy (≥40 PY)	153,145 (3.7)	16,096 (8.2)	65,922 (3.7)	7,015 (8.4)
Alcohol consumption				
Non-drinker	2,417,968 (57.9)	122,179 (62.4)	1,036,246 (57.9)	52,404 (62.4)
Mild (<15g/day)	1,031,629 (24.7)	39,427 (20.2)	441,972 (24.7)	16,795 (20.0)
Moderate (≥15g/day, <30g/day)	417,518 (10.0)	17,944 (9.2)	179,142 (10.0)	7,698 (9.2)
Heavy (≥30g/day)	306,872 (7.4)	16,146 (8.3)	131,433 (7.4)	7,031 (8.4)
Physical activity				
None	2,080,550 (49.9)	113,255 (57.9)	890,644 (49.8)	48,279 (57.5)
Insufficient	1,257,052 (30.1)	45,760 (23.4)	538,955 (30.1)	19,826 (23.6)
Sufficient	836,385 (20.0)	36,681 (18.7)	359,194 (20.1)	15,823 (18.9)
Diabetes				
Normal (FPG<100mg/dL)	2,682,620 (64.3)	103,715 (53.0)	1,148,466 (64.2)	44,186 (52.7)
Prediabetes (100≤FPG<126mg/dL)	1,060,020 (25.4)	49,405 (25.3)	455,300 (25.5)	21,684 (25.8)
Untreated diabetes (FPG≥126mg/dL)	145,608 (3.5)	10,170 (5.2)	62,331 (3.5)	4,360 (5.2)
Recent onset diabetes (<5 years)	152,559 (3.7)	14,325 (7.3)	65,571 (3.7)	5,975 (7.1)
Long-standing diabetes (≥5 years)	133,180 (3.2)	18,081 (9.2)	57,125 (3.2)	7,723 (9.2)
Hypertension				
Normal	1,279,977 (30.7)	32,800 (16.8)	547,098 (30.6)	14,265 (17.0)
Prehypertension	1,549,316 (37.1)	57,157 (29.2)	664,264 (37.1)	24,800 (29.6)
New-onset hypertension	389,178 (9.3)	22,955 (11.7)	166,806 (9.3)	9,566 (11.4)
Controlled with anti-hypertensives	631,400 (15.1)	52,578 (26.9)	271,745 (15.2)	22,189 (26.4)
Uncontrolled with anti-hypertensives	324,116 (7.8)	30,206 (15.4)	138,880 (7.8)	13,108 (15.6)
Dyslipidemia				
Untreated, TC<200mg/dL	2,006,530 (48.1)	82,055 (41.9)	860,711 (48.1)	35,026 (41.7)
Untreated, 200mg/dL≤TC<240mg/dL	1,277,983 (30.6)	57,647 (29.5)	546,818 (30.6)	24,832 (29.6)
Untreated, 240mg/dL≤TC	405,514 (9.7)	22,018 (11.3)	173,551 (9.7)	9,449 (11.3)
Treated with lipid-lowering agent	483,960 (11.6)	33,976 (17.4)	207,713 (11.6)	14,621 (17.4)
CKD (estimated GFR)				
<30	4,358 (0.1)	684 (0.4)	1,815 (0.1)	298 (0.4)
30-60	226,193 (5.4)	22,199 (11.3)	97,027 (5.4)	9,353 (11.1)
≥60	3,943,436 (94.5)	172,813 (88.3)	1,689,951 (94.5)	74,277 (88.5)
Family history of stroke or MI	384,320 (9.2)	16,080 (8.2)	164,222 (9.2)	6,812 (8.1)
Systolic blood pressure	123.69±15.37	129.18±16.63	123.71±15.35	129.15±16.72
Diastolic blood pressure	77.01±10.2	79.19±10.59	77.01±10.2	79.14±10.56
Fasting glucose, mg/dL	99.25±24.75	106.89±36.27	99.27±24.71	106.94±36.33
Total cholesterol, mg/dL	199.38±36.92	202.12±39.63	199.35±36.9	202.28±39.61
Creatinine, serum, mg/dL	0.9±0.22	0.93±0.26	0.9±0.22	0.93±0.26
Follow-up duration, years	9.17±1.14	5.35±2.69	9.17±1.14	5.35±2.7

CVD, cardiovascular disease; PY, pack*year; FPG, fasting plasma glucose; TC, total cholesterol; CKD, chronic kidney disease; GFR, glomerular filtration rate; MI, myocardial infarction

eTable S4 Predictors of cardiovascular disease in the risk prediction model

	Subjects (N)	Cases (n)	IR	Crude Model HR (95% CI)	Final Model aHR (95% CI)	Point
Age, years						
40-44	958,910	13,326	1.51	1 (Ref.)	1 (Ref.)	0
45-49	801,224	17,018	2.31	1.53 (1.50-1.57)	1.45 (1.41-1.48)	14
50-54	862,220	25,623	3.24	2.15 (2.11-2.20)	1.95 (1.91-1.99)	25
55-59	511,152	22,012	4.75	3.16 (3.09-3.23)	2.65 (2.60-2.71)	37
60-64	495,753	29,624	6.66	4.43 (4.34-4.52)	3.57 (3.50-3.65)	48
65-69	329,690	29,803	10.40	6.96 (6.81-7.10)	5.37 (5.25-5.48)	63
70-74	267,354	34,342	15.48	10.44 (10.23-10.65)	7.91 (7.75-8.08)	78
75-79	92,800	15,309	21.65	14.87 (14.53-15.22)	11.26 (10.98-11.54)	91
≥80	50,580	8,639	26.46	18.86 (18.36-19.38)	14.19 (13.79-14.60)	100
Male, sex	2,164,819	110,491	5.75	1.36 (1.35-1.37)	1.34 (1.32-1.36)	11
Female, sex	2,204,864	85,205	4.24	1 (Ref.)	1 (Ref.)	0
Body mass index, kg/m^2						
<18.5	99,200	5,371	6.38	1.45 (1.41-1.49)	1.19 (1.16-1.22)	7
18.5-23	1,598,569	63,988	4.45	1 (Ref.)	1 (Ref.)	0
23-25	1,163,819	51,389	4.89	1.10 (1.08-1.11)	0.99 (0.98-1.00)	0
25-30	1,366,503	67,315	5.47	1.23 (1.21-1.24)	1.02 (1.01-1.03)	1
≥30	141,592	7,633	6.00	1.35 (1.32-1.38)	1.11 (1.08-1.13)	4
Smoking behavior						
Non-smoker	2,807,429	116,286	4.57	1 (Ref.)	1 (Ref.)	0
Mild (<20 PY)	806,675	31,198	4.30	0.94 (0.93-0.95)	1.18 (1.17-1.20)	6
Moderate (≥20 PY, <40 PY)	586,338	32,116	6.18	1.36 (1.34-1.38)	1.45 (1.43-1.47)	14
Heavy (≥40 PY)	169,241	16,096	11.31	2.51 (2.46-2.55)	1.58 (1.55-1.61)	17
Alcohol consumption						
Non-drinker	2,540,147	122,179	5.35	1 (Ref.)	1 (Ref.)	4
Mild (<15g/day)	1,071,056	39,427	4.06	0.76 (0.75-0.77)	0.89 (0.88-0.90)	0
Moderate (≥15g/day, <30g/day)	435,462	17,944	4.58	0.86 (0.84-0.87)	0.91 (0.89-0.92)	1
Heavy (≥30g/day)	323,018	16,146	5.63	1.05 (1.04-1.07)	0.97 (0.95-0.99)	3
Physical activity						
None	2,193,805	113,255	5.78	1 (Ref.)	1 (Ref.)	7
Insufficient	1,302,812	45,760	3.87	0.67 (0.66-0.67)	0.86 (0.85-0.87)	2
Sufficient	873,066	36,681	4.65	0.80 (0.79-0.81)	0.82 (0.81-0.83)	0
Diabetes						
Normal (FPG<100mg/dL)	2,786,335	103,715	4.10	1 (Ref.)	1 (Ref.)	0
Prediabetes (100≤FPG<126mg/dL)	1,109,425	49,405	4.96	1.21 (1.20-1.22)	1.00 (0.99-1.01)	0
Untreated diabetes (FPG≥126mg/dL)	155,778	10,170	7.47	1.84 (1.80-1.87)	1.35 (1.32-1.38)	11
Recent onset diabetes (<5 years)	166,884	14,325	9.92	2.43 (2.39-2.47)	1.46 (1.43-1.49)	14
Long-standing diabetes (≥5 years)	151,261	18,081	14.26	3.52 (3.47-3.58)	1.82 (1.79-1.86)	23
Hypertension						
Normal	1,312,777	32,800	2.73	1 (Ref.)	1 (Ref.)	0
Prehypertension	1,606,473	57,157	3.92	1.44 (1.42-1.46)	1.18 (1.16-1.19)	6
New-onset hypertension	412,133	22,955	6.28	2.31 (2.27-2.35)	1.53 (1.51-1.56)	16
Controlled with anti-hypertensives	683,978	52,578	8.79	3.24 (3.20-3.29)	1.50 (1.48-1.52)	15
Uncontrolled with anti-hypertensives	354,322	30,206	9.80	3.61 (3.56-3.67)	1.61 (1.59-1.64)	18
Dyslipidemia						
Untreated, TC<200mg/dL	2,088,585	82,055	4.37	1 (Ref.)	1 (Ref.)	0
Untreated, 200mg/dL≤TC<240mg/dL	1,335,630	57,647	4.78	1.09 (1.08-1.11)	1.08 (1.07-1.10)	3
Untreated, 240mg/dL≤TC	427,532	22,018	5.74	1.32 (1.30-1.34)	1.28 (1.26-1.30)	9
Treated with lipid-lowering agent	517,936	33,976	7.35	1.68 (1.66-1.70)	1.03 (1.02-1.05)	1
CKD (Estimated GFR)						
<30	5,042	684	17.82	3.93 (3.65-4.24)	2.14 (1.98-2.31)	29
30-60	248,392	22,199	10.44	2.26 (2.23-2.29)	1.19 (1.17-1.21)	7
≥60	4,116,249	172,813	4.65	1 (Ref.)	1 (Ref.)	0
Family history of stroke or MI						
No	3,969,283	179,616	5.04	1 (Ref.)	1 (Ref.)	0
Yes	400,400	16,080	4.42	0.88 (0.86-0.89)	1.04 (1.02-1.06)	1

HR, hazard ratio; aHR, adjusted hazard ratio; PY, pack*year; FPG, fasting plasma glucose; TC, total cholesterol; CKD, chronic kidney disease; GFR, glomerular filtration rate; MI, myocardial infarction

SECTION S2. Supplemental Methods

eMethod S1. Classification of each predictors

Age was divided into 5-year intervals (40–44, 45–49, 50–54, 55–59, 60–64, 65–69, 70–74, 75–79, ≥80). BMI (kg/m^2) was divided into five groups (<18.5, 18.5–23, 23–25, 25–30, ≥30) according to the Asian-Pacific criteria.[1] Smoking habits were categorized by pack-years (PYs) as none, mild (less than 20 PYs), moderate (20 to less than 40 PYs), and heavy (40 or more PYs). Alcohol consumption was categorized by grams per day as none, mild (less than 15 grams per day), moderate (15 to less than 30 grams per day), and heavy (30 or more grams per day).[2] Physical activity was categorized as none, insufficient, or sufficient. Sufficient physical activity was defined as moderate-intensity exercise performed at least five times per week for a minimum of 30 minutes per session or high-intensity exercise performed three times per week for a minimum of 20 minutes per session.

The assessments of diabetes, hypertension, and dyslipidemia were based on participants' past medical history and ICD-10 codes from clinical and pharmacy records. For diabetes status at baseline, participants were classified into five categories according to their glycemic status[3]: (1) normal (FPG [fasting plasma glucose] <100mg/dL]), (2) prediabetes (FPG 100–125mg/dL), (3) new-onset diabetes (FPG ≥126mg/dL without diagnosis of diabetes), (4) recent onset diabetes (duration less than 5 years), and (5) long-standing diabetes (duration 5 years or longer). A diagnosis of diabetes during follow up was identified by ICD-10 codes (E11.x-E14.x) with antidiabetic medications or an FPG ≥ 126 mg/dL, which allowed classification into (3), (4), or (5).

Hypertension was classified into five groups by systolic/diastolic blood pressure (SBP/DBP), and the use of anti-hypertensives:[4] (1) normal (SBP <120mmHg, DBP <80mmHg); (2) prehypertension (undiagnosed, SBP 120–140mmHg or DBP 80–90mmHg); (3) new-onset hypertension (SBP ≥140mmHg or DBP ≥90mmHg); (4) controlled with anti-

hypertensives (SBP <140mmHg, DBP <90mmHg); and (5) uncontrolled with anti-hypertensives (SBP ≥140mmHg or DBP ≥90mmHg). Dyslipidemia was stratified into four categories: (1) untreated, total cholesterol <200mg/dL; (2) untreated, total cholesterol 200–240mg/dL; (3) untreated, total cholesterol ≥240mg/dL; and (4) treated with lipid-lowering agents. Chronic kidney disease was determined based on the glomerular filtration rate (GFR) estimated by the Modification of Diet in Renal Disease equation. The estimated GFR was classified as ≥60, 30–60, and <30 ml/min/1.73 m^2. The family history of CVD was obtained through a questionnaire self-administered during the general health examination. Using a yes/no question, participants were asked to report whether any of their first-degree relatives (parents or siblings) had experienced a stroke or MI.

SECTION S3. eReference

1. World Health Organization. Regional Office for the Western P. *The Asia-Pacific perspective : redefining obesity and its treatment*. Sydney : Health Communications Australia; 2000.
2. Jeong S-M, Lee HR, Han K, et al. Association of Change in Alcohol Consumption With Risk of Ischemic Stroke. *Stroke*. 2022;53(8):2488-2496. doi:doi:10.1161/STROKEAHA.121.037590
3. Yoo JE, Kim D, Han K, Rhee SY, Shin DW, Lee H. Diabetes Status and Association With Risk of Tuberculosis Among Korean Adults. *JAMA Netw Open*. Sep 1 2021;4(9):e2126099. doi:10.1001/jamanetworkopen.2021.26099
4. Kim YG, Han K-D, Choi J-I, et al. Impact of the Duration and Degree of Hypertension and Body Weight on New-Onset Atrial Fibrillation. *Hypertension*. 2019;74(5):e45-e51. doi:doi:10.1161/HYPERTENSIONAHA.119.13672
