# Supplementary figures and images for: Separating Risk Prediction: Myocardial Infarction vs. Ischemic Stroke in 6.2M Screenings
Source: Healthcare (Basel). 2024 Oct 18;12(20):2080. doi: 10.3390/healthcare12202080 (PMC11507110; doi:10.3390/healthcare12202080)

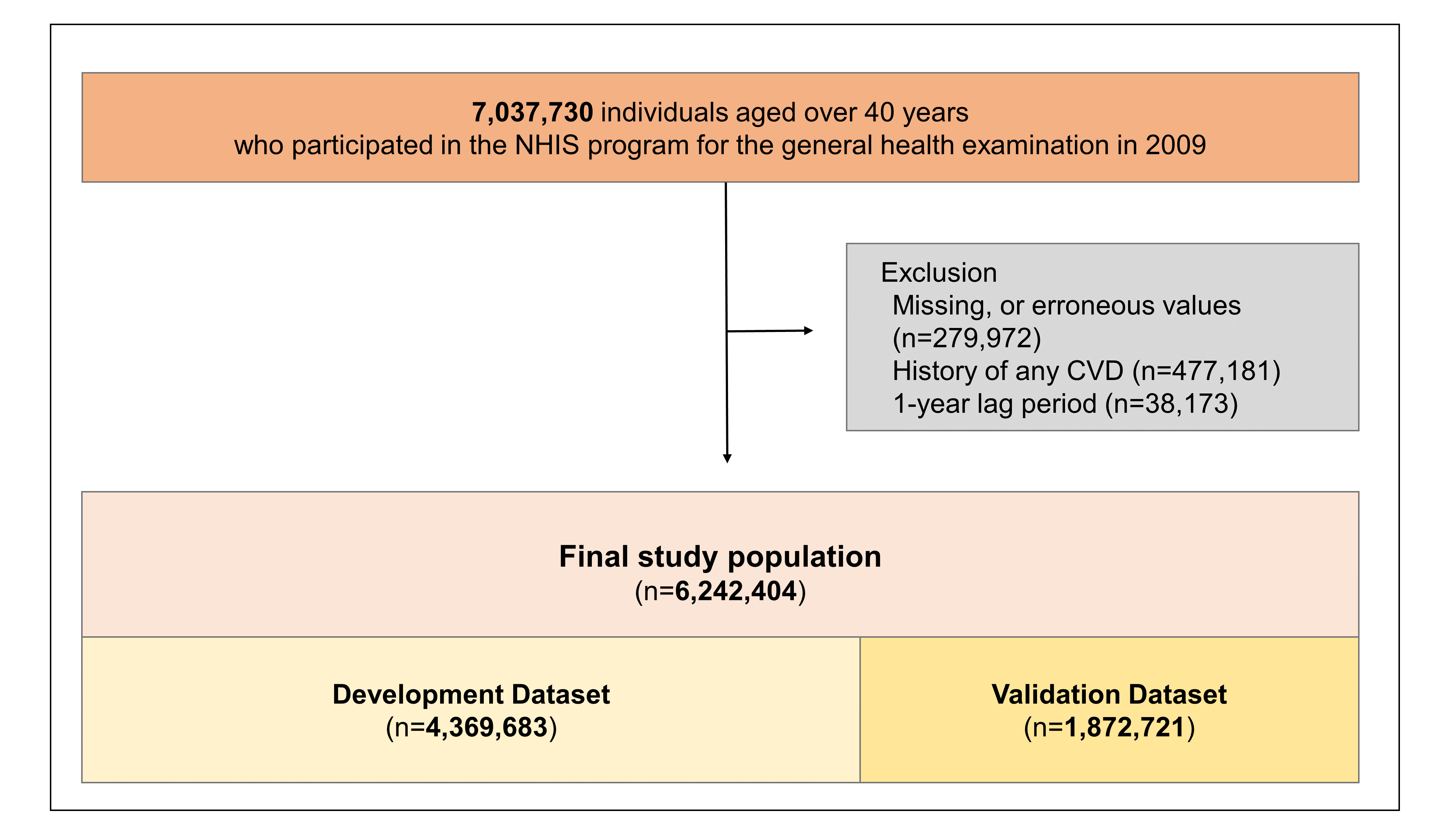

Supplement: Supplementary file 1 [file healthcare-12-02080-s001.zip › eFigureS1.PNG]

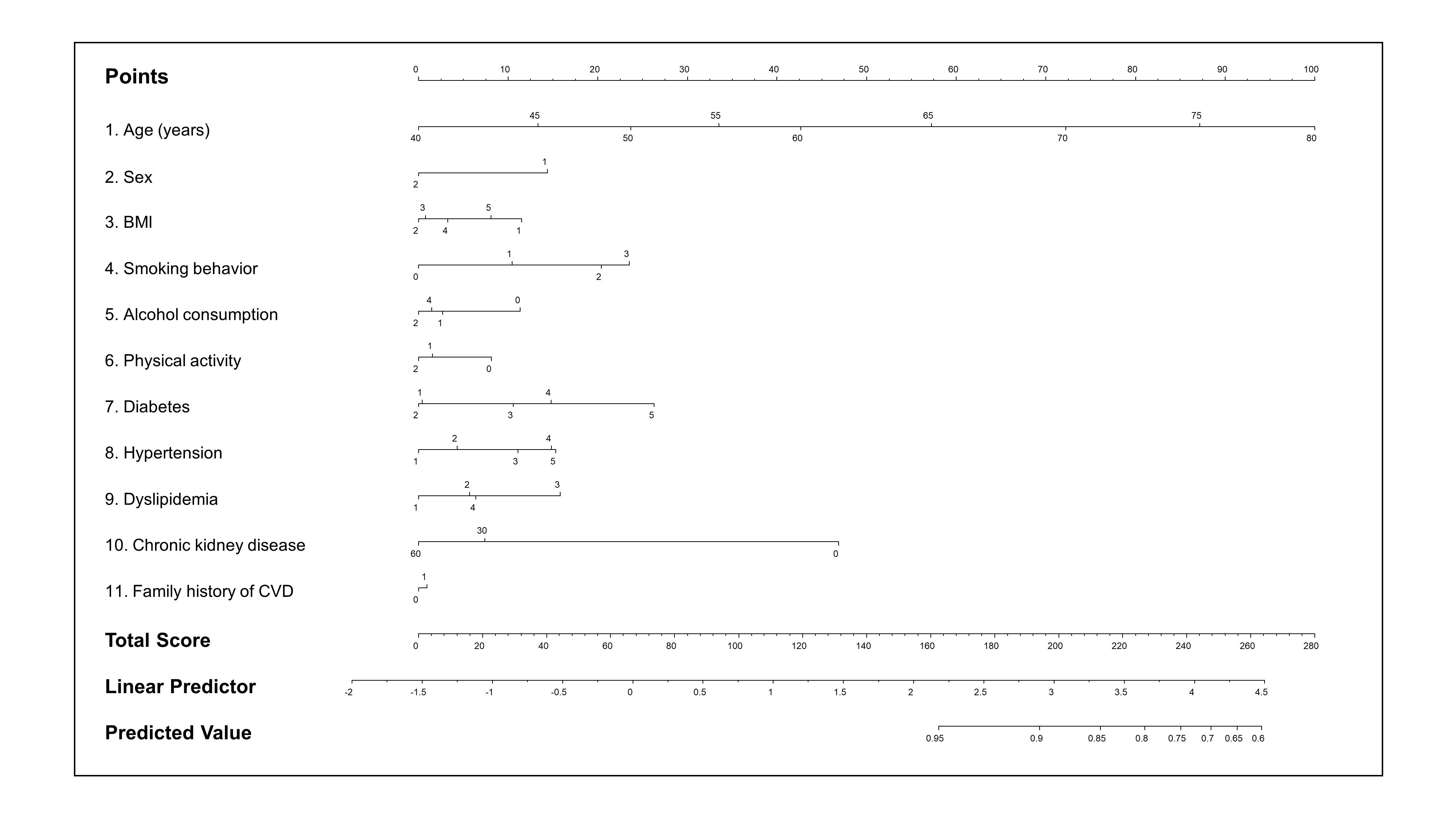

Supplement: Supplementary file 1 [file healthcare-12-02080-s001.zip › eFigureS2.PNG]

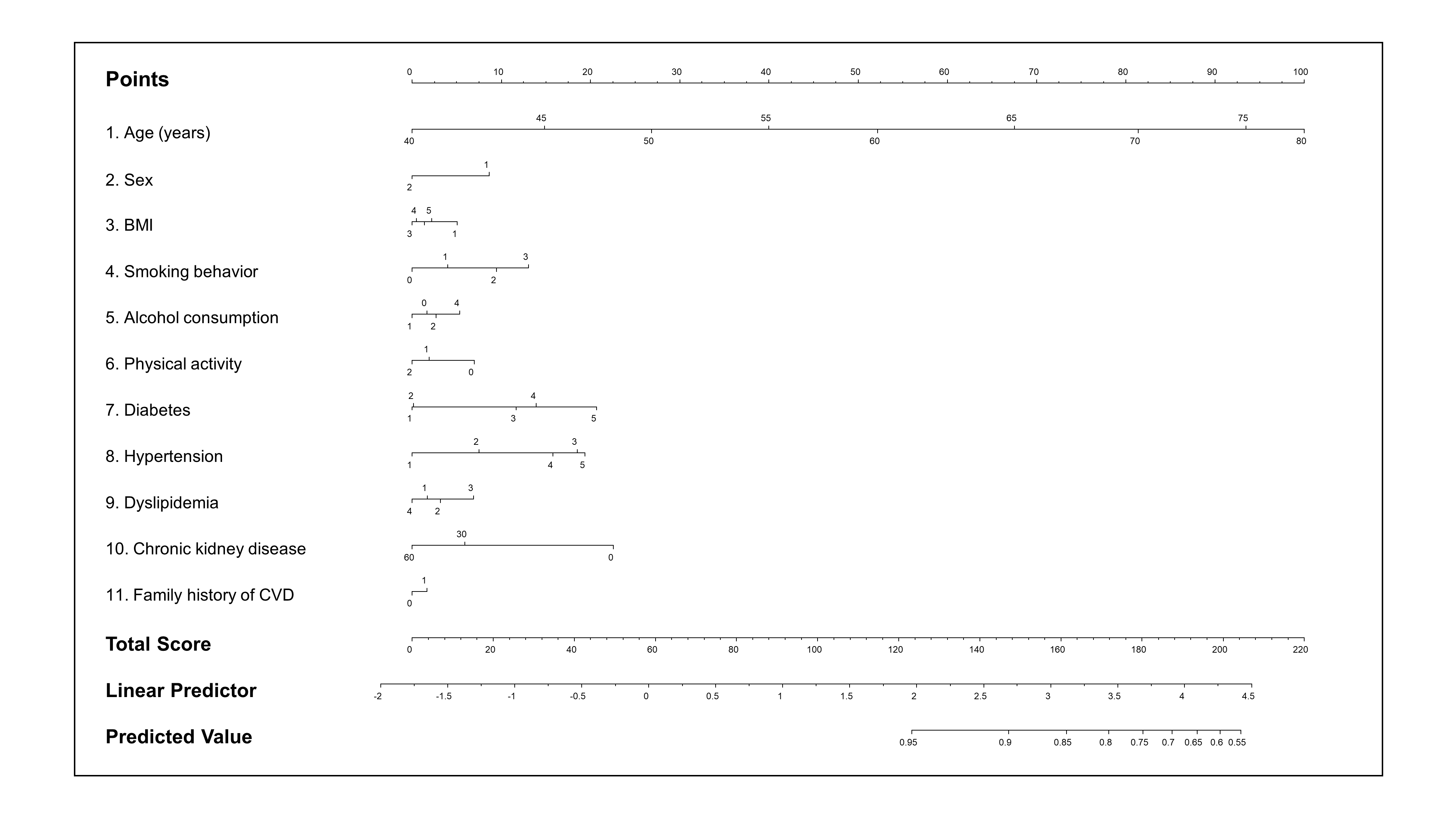

Supplement: Supplementary file 1 [file healthcare-12-02080-s001.zip › eFigureS3.PNG]

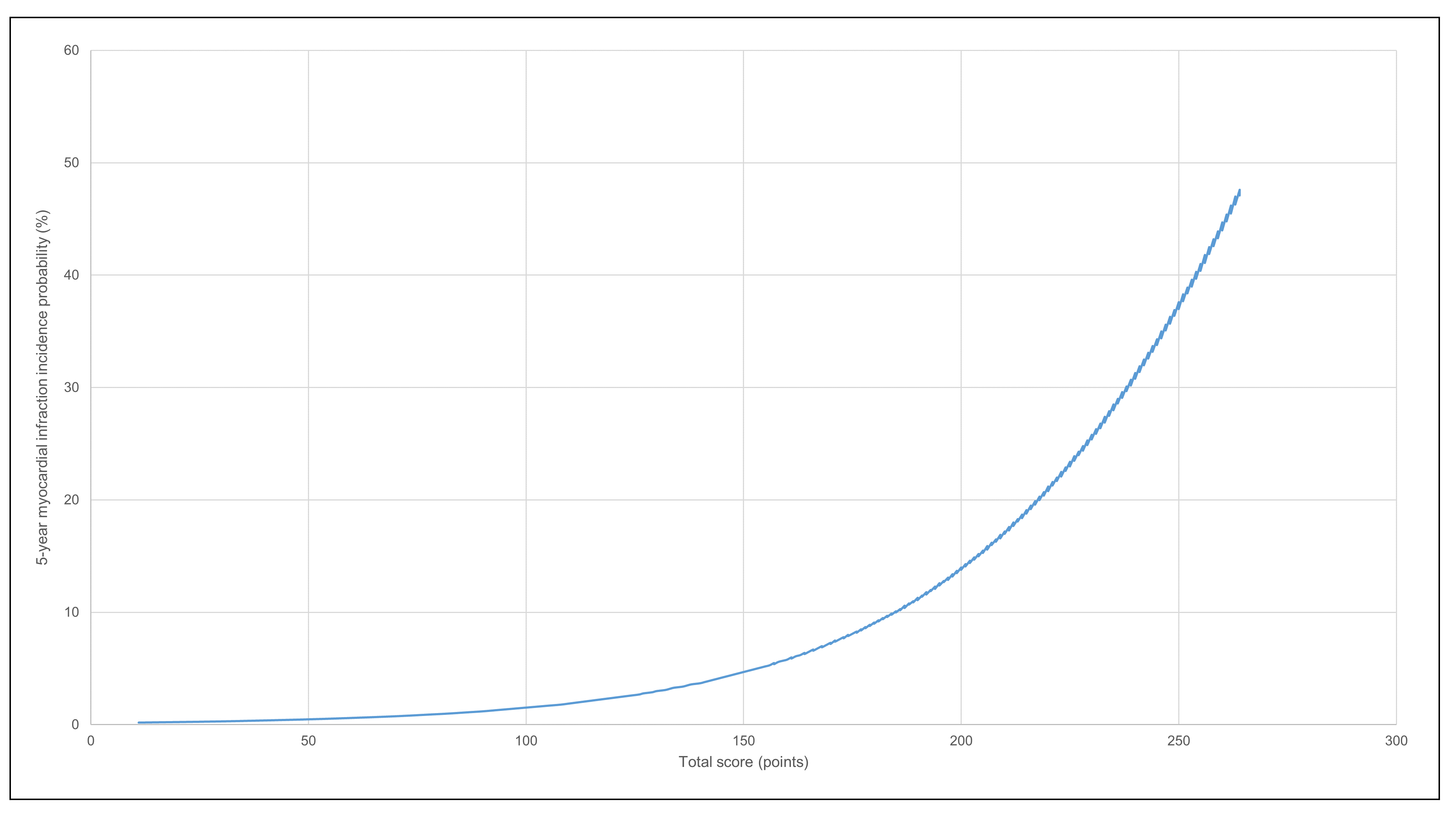

Supplement: Supplementary file 1 [file healthcare-12-02080-s001.zip › eFigureS4.PNG]

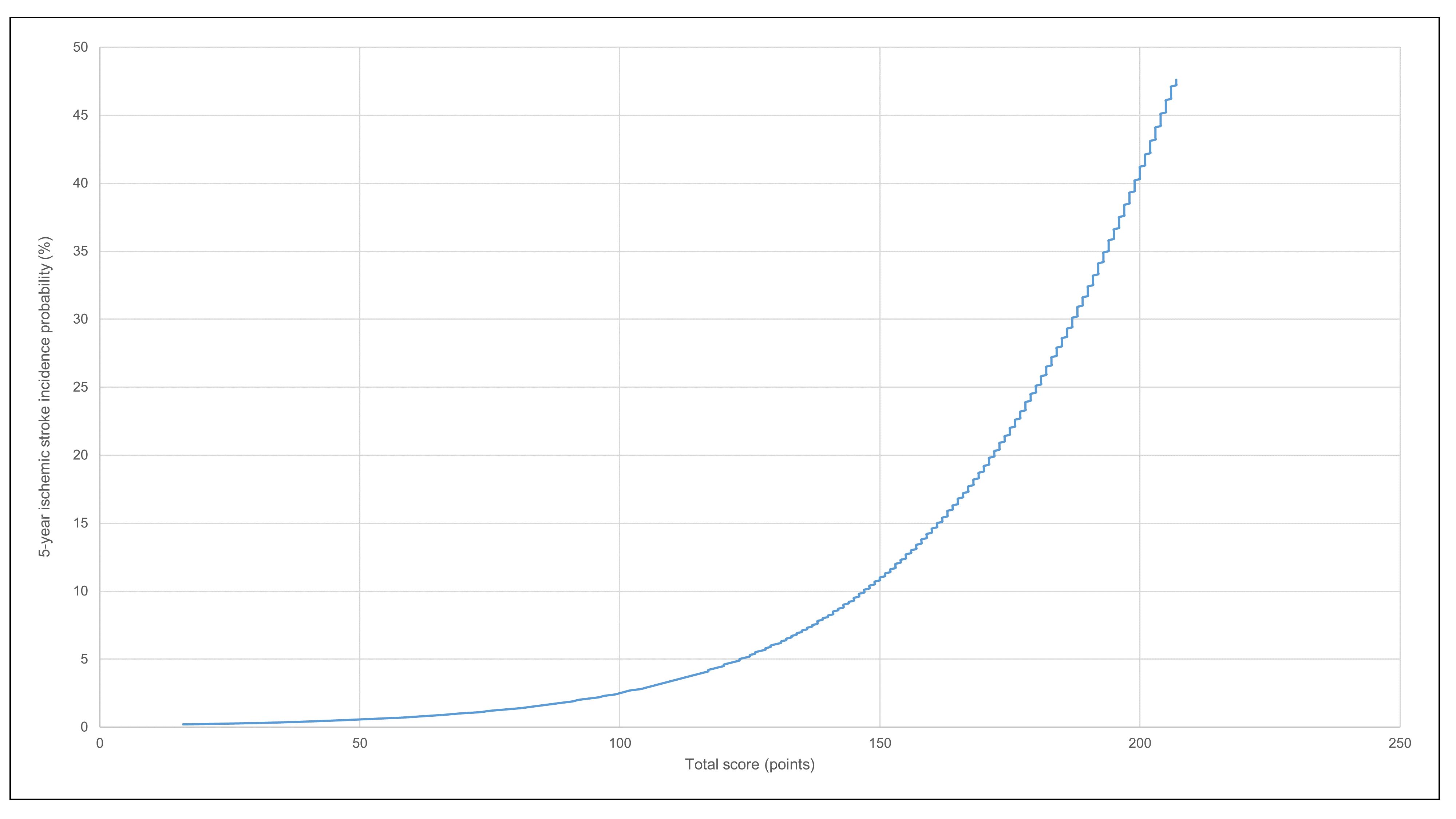

Supplement: Supplementary file 1 [file healthcare-12-02080-s001.zip › eFigureS5.PNG]

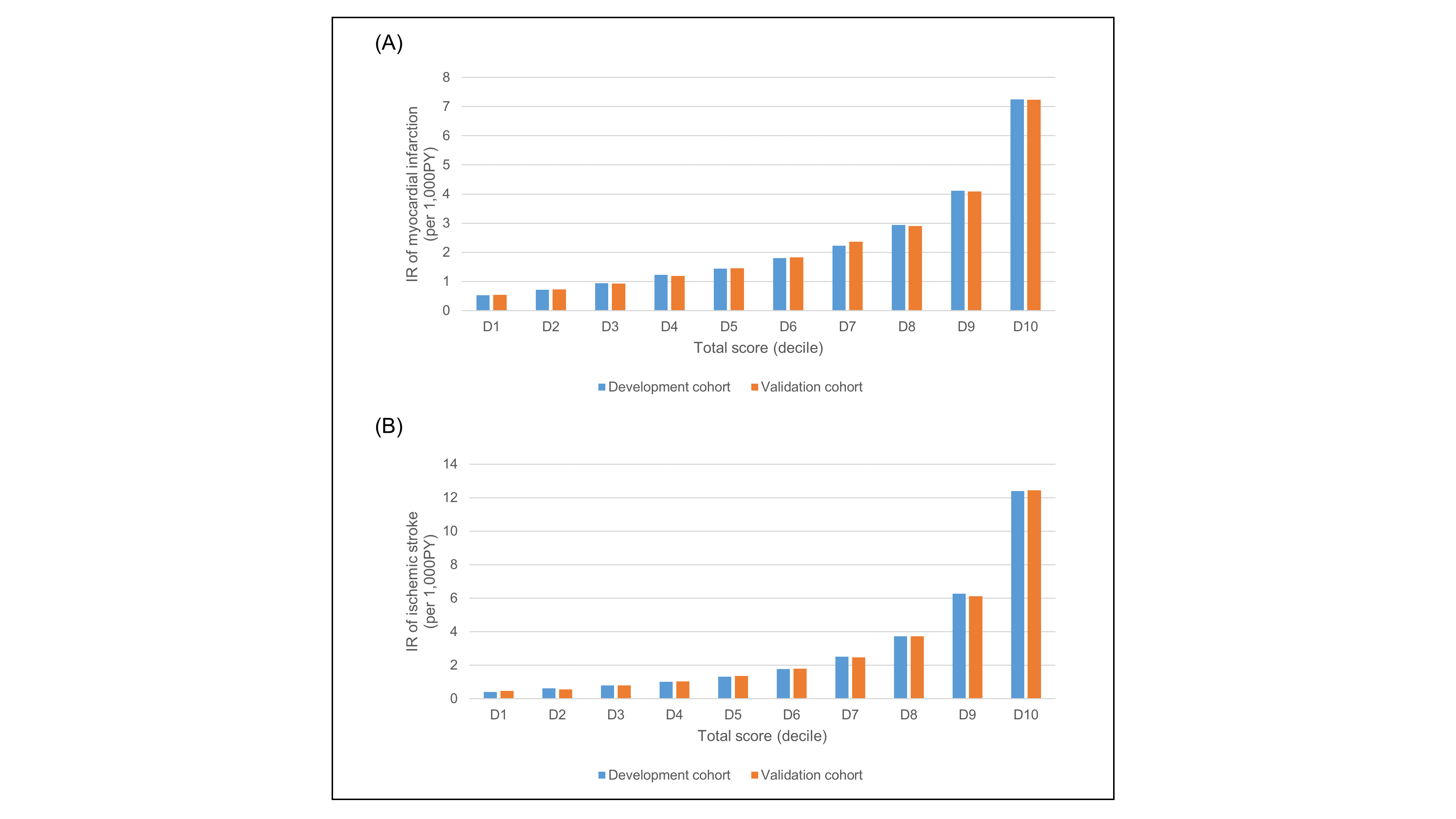

Supplement: Supplementary file 1 [file healthcare-12-02080-s001.zip › eFigureS6.PNG]

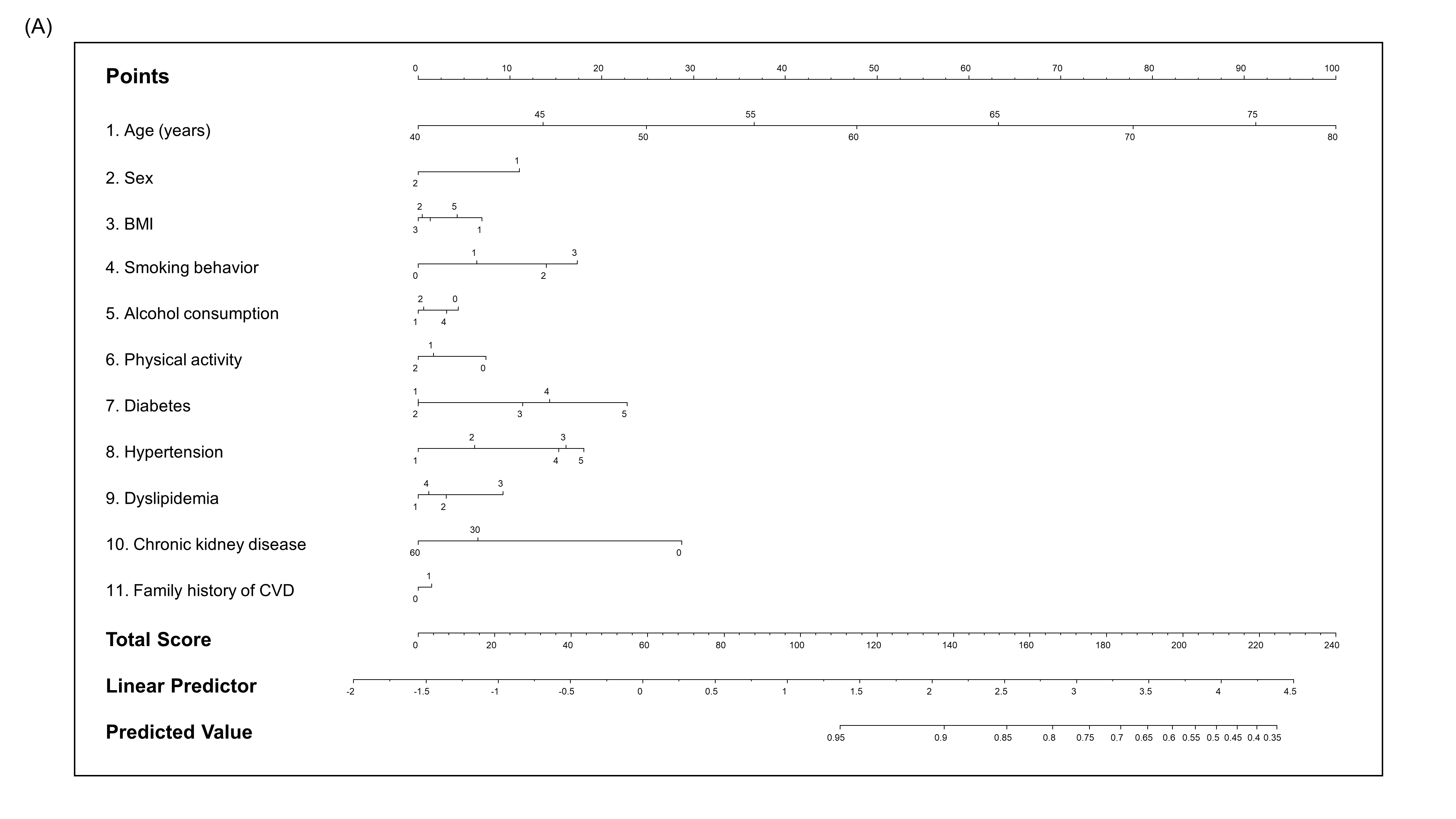

Supplement: Supplementary file 1 [file healthcare-12-02080-s001.zip › eFigureS7.PNG]

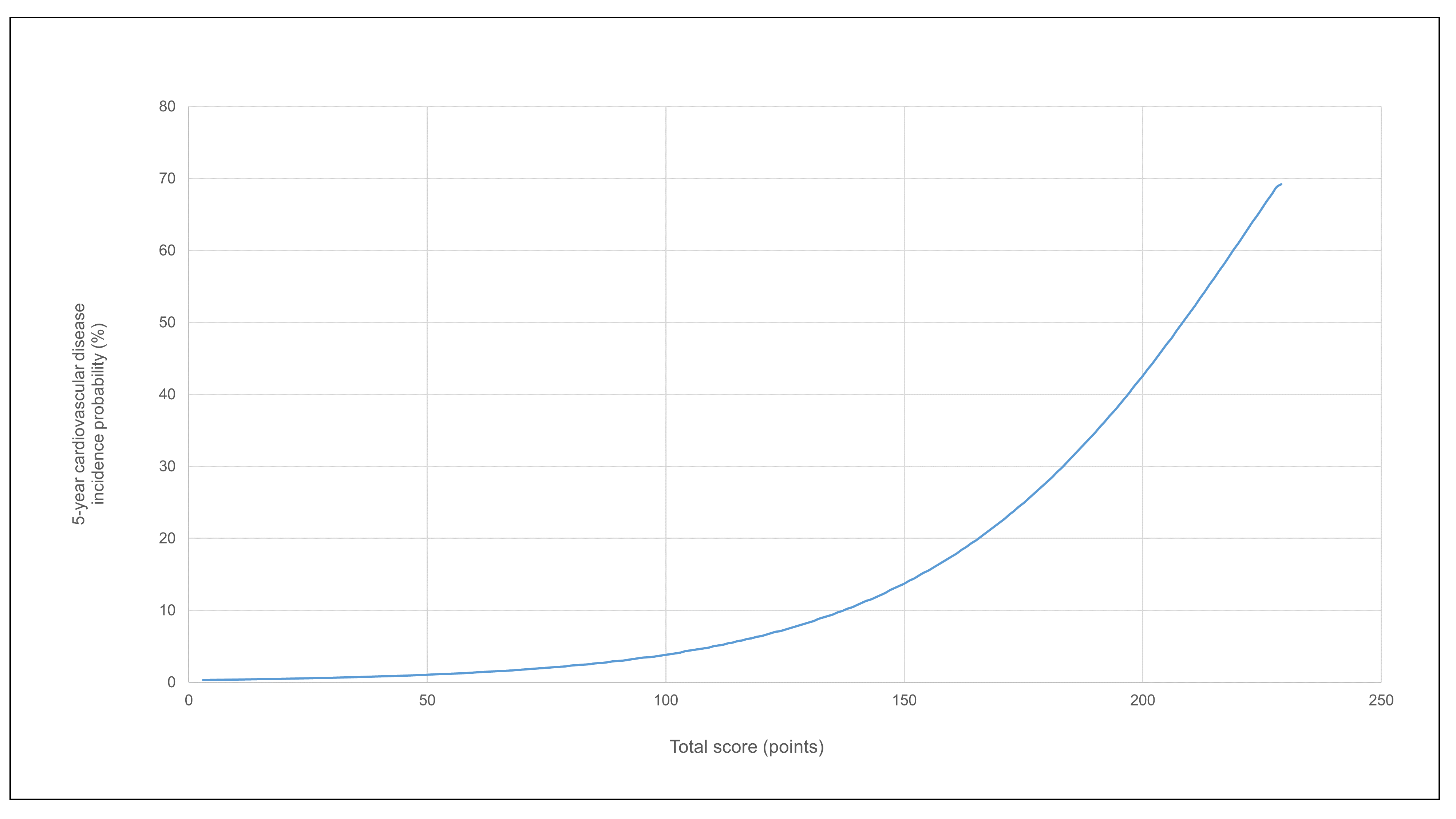

Supplement: Supplementary file 1 [file healthcare-12-02080-s001.zip › eFigureS8.PNG]

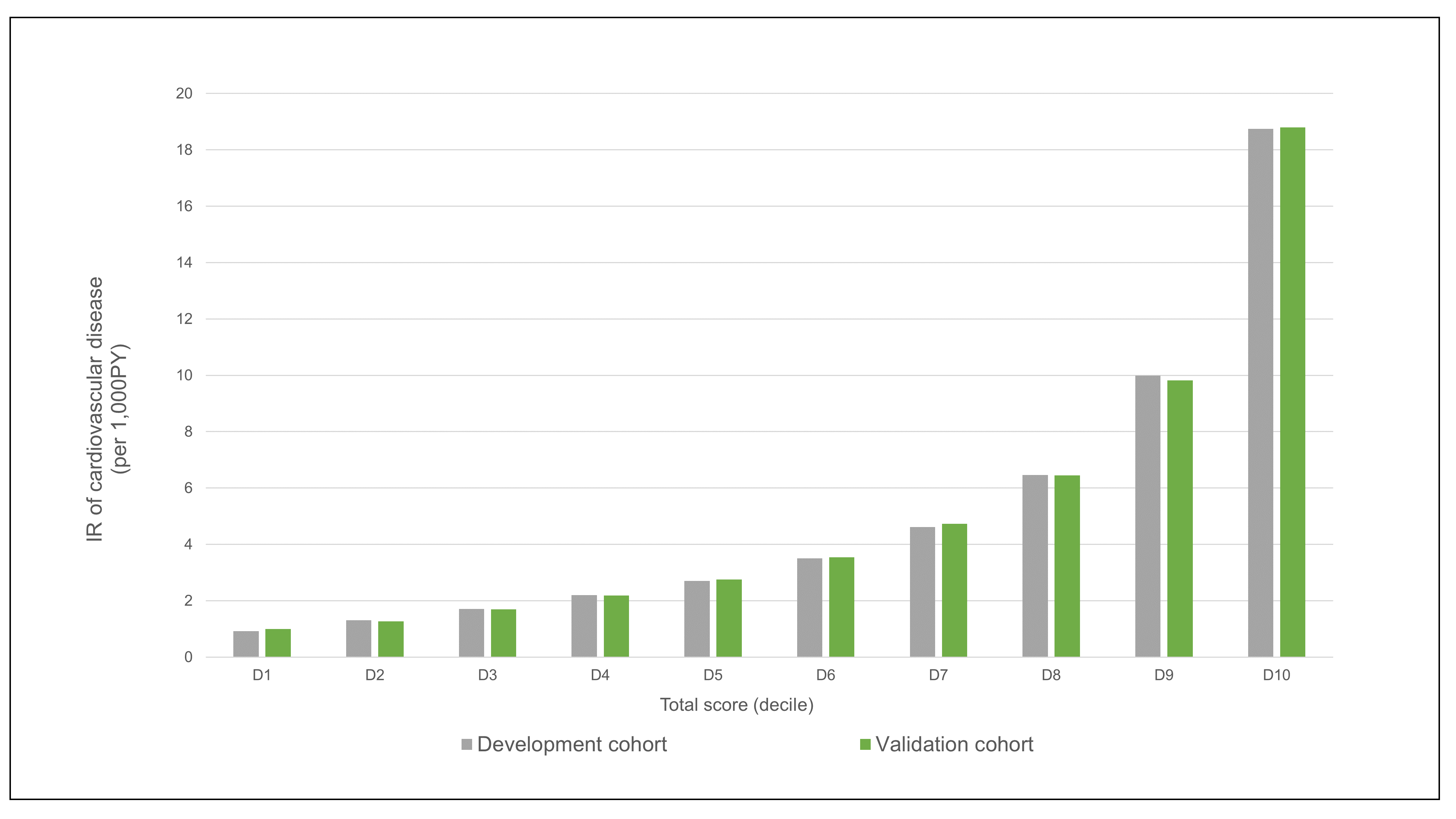

Supplement: Supplementary file 1 [file healthcare-12-02080-s001.zip › eFigureS9.PNG]
